# 

**Published:** 1853-01

**Authors:** 


### CONTENTS.

### JOURNAL.

Article I.—On the Treatment of Deep Seated Dental Caries. By W. H. Dwinelle, M. D., D. D. S. . . . . . . 169

Article II.—Proposition for Extending the Requirements of a Collegiate Dental Education, so as to embrace a Course of Lectures in a Medical School proper. By B. Wood, M. D. 180

Article III.—Review of Drs. Gardette and Trenor, on Dental Colleges and Dental Education. By W. W. H. Thackston, M. D., D. D. S. . . . . . . . . . . 190

Article IV.—Anatomy and Physiology of Dentine, an Essay read before the American Society of Dental Surgeons, convened at Newport, R. I., August, 1852. By J. De Haven White, M. D., D. D. S. . . . . . . . . . . . 215

Article V.—The Replacement of the Teeth, with a Plan for their Retention. By C. T. Cushman, D. D. S. . . . . 224

Article VI.—Ophthalmitis, from Wearing a Dental Substitute not Properly Adjusted. By H L. Burpee, Suffield, Conn. . 232

Article VII.—Microscopes of Large Angle of Aperture. By W. H. Dwinelle, M. D., D. D. S. . . . . . . . 236

Article VIII.—Gold, Gold-beating, Compounds and Alloys. By R. N. Wright, M. D. . . . . . . . . . . . 243

Article IX.—Chemistry and its application to Dentistry. By A. Snowden Piggot, M. D. . . . . . . . . . 248

Article X.—On the Large Deposits of Salivary Calculus in Gouty Persons. By J. L. Levison, D. D. S. . . . . . . 256

Article XI.—Insertion of Plate Teeth, on a Pivot. By S. A. Saltenstall, D. D. S. . . . . . . . . . . 258

Article XII.—Patents and Patentees, By J. W. Keyes, M. D., D. D. S. . . . . . . . . . . . . 259

### SELECTED ARTICLES.

Article XIII.—Observations on Excision of the Superior Maxillary Bone. By S. D. Gross, M. D. . . . . . . 262

Article XIV.—Review of Dr. Hunter's third set of Formulas. By John Allen, D. D. S. . . . . . . . . . . 280

### *Contents.*

ARTICLE XV.—Case of Carcinoma of the Eyelid. By C. Theodor Meier, M. D. . . . . . . . . . . . 290
ARTICLE XVI.—Case of Crancum Oris, with Necrosis of a large portion of the Inferior Maxillary Bone, followed by Recovery. By T. Herbert Barker, M. D., F. R. C. S. . . . . . 293
ARTICLE XVII.—Retro-Pharyngeal Abscess—Its Medical History and Treatment. By Charles M. Allin, M. D. . . . 297
ARTICLE XVIII.—Remarks upon making Gold Plate and Solder. By B. Wood, M. D., Dentist, . . . . . . . . 308
ARTICLE XIX.—Wound of the Skull, . . . . . . 314
ARTICLE XX.—Scirrhus of the Sub-Maxillary Gland, . . 315

### CORRESPONDENCE.

A Few Weeks Abroad—Notes by the Way. By W. H, Dwinelle, M. D., D. D. S. . . . . . . . . . . 317

### BIBLIOGRAPHICAL.

Die Zahnheilkunde, nach ihrem neusten Standpunkte, ein Lehibuck für Zahraerzte unt Aerzte, von J. Liuderer, Zahnarzt in Berlin, mit 6 tafeln in Stahlstich, . . . . . . . 323
Discovery by the late Horace Wells of the Applicability of Nitrous Oxyd Gas, Sulphuric Ether and other Vapors, in Surgical Operations, . . . . . . . . . . . . 323
Wythe's Pocket Dose and Symptom Book, . . . . . . 324
Carpenter's Principles of Human Physiology, . . . . . 324
The Druggists' General Receipt Book, . . . . . . . 325
Report on Morton's Claim of the Discovery of Anæsthetic Agents, 325
The Mirror of Dentistry, . . . . . . . . . . 325

### EDITORIAL DEPARTMENT.

A word to our Exchanges. . . . . . . . . . 327
Dr. B. Wood on Dental Education, . . . . . . . . 331
Osseous Union of Teeth, . . . . . . . . . . . 333
Ancient Dental Instruments, . . . . . . . . . . 333
A New Year, . . . . . . . . . . . . . 334
New Jersey Medical Institute, located at Burlington, N. J., . . . 334
Virginia Medical and Surgical Journal, . . . . . . . 335
Movable Seat Dentist's Chair, . . . . . . . . . 335
Obituary, . . . . . . . . . . . . . . 335

